# The Hospital Officer's Bookshelf

**Published:** 1912-11-30

**Authors:** 


### THE HOSPITAL OFFICER'S BOOKSHELF.

Manual of Human Embryology. By numerous authors; edited by Franz Keibel and Franklin P. Moll. Vol. II. (Lippincott Company. 1912. Pp. 1032. £2 net.)

The concluding volume of this truly monumental and magnificent work suffers under the disadvantage of a somewhat delayed publication. But embryologists will be too thankful to have it at last to grumble very much at the delay, so often inevitable when a great number of collaborators have to be employed. The contents of this second volume deal in detail with the embryology of the nervous system, the sense organs, the digestive tract, respiratory organs, blood-vascular system, and urogenital system. To deal in any but general terms with these subjects would require many pages of this journal; and to offer occasional criticisms upon minor points which seem to be open to objection would be to risk giving a wrong idea of the general quality of the work. Suffice it to say that no stone has been left unturned by editors and staff to furnish a complete and accurate account of everything that is known about embryology. The progress of research will doubtless make—nay, is already making—this book in the end an out-of-date manual; but at the present day it represents the last word in embryological text-books, and is indispensable to everyone who desires to master that rapidly expanding science.

The Surgery of the Skull and Brain. By L. Bathe Rawling, F.R.C.S. Lond. (London: Henry Frowde and Hodder and Stoughton, Warwick Square, E.C. 1912. Price 25s. net.)

It is somewhat difficult to fathom what is the object of this book, which is one of the admirable Oxford Medical Series. If Mr. Rawling writes for the specialist, for the surgeon who is capable of undertaking a case of brain surgery—as most general surgeons are—his book is largely a work of supererogation, for he gives us nothing new, and his compilation lacks the clearness which characterises many old-fashioned text-book descriptions, such as for instance the chapters dealing with this subject in Bergmann's and von Brun's books or the good article on brain surgery in Jacobson and Steward's "Operations of Surgery." When we compare his work with special monographs such as are found in the text-books of the Deutsche Chirurgie Series or in the lists of French and Italian publishers, we must confess that we cannot honestly say he has succeeded in "representing the modern aspects of the case." The book is not up to date as regards the literature; and no bibliography is added. As an instance, we may cite the bald treatment of so important a subject as the tumours and pathology of the pituitary body, in which no foreign authorities are quoted. If, on the other hand, Mr. Rawling intends his book for the student, it has obvious merits, for it is short and concise, but in that case we are afraid its price must seriously militate against its success.

Practical Anatomy: An Exposition of the Facts of Gross Anatomy from the Topographical Standpoint, and a Guide to the Dissection of the Human Body. By John C. Heisler, M.D. (Philadelphia and London: J. B. Lippincott Company. Price 21s. net.)

We have read this one-volume manual of practical anatomy with much interest. While it lacks the fulness and well-ordered arrangement that were such conspicuous features of the popular old Ellis manual (now, alas! we believe, metamorphosed into a double volume), it is much more concise and handy than the well-known Cunningham. It is excellently illustrated, possessing no fewer than 366 cuts, of which 225 are in colours, many of them derived from familiar text-books. In the introduction good practical hints are given; the photographs showing the methods used in the dissecting-room are admirable. The descriptive part is lucid and clear, though we must admit that we prefer Cunningham's way of dealing with muscles and their attachments. Nor do we see any reason to introduce pathology or the finer details of surgical anatomy into a manual which is obviously destined for a beginner. The information is sound and the various dissections are well handled, but we do not anticipate that the book will find much favour with English students who are acquainted with Ellis and Cunningham. For those who want a single-volume manual for revision purposes it is admirably suited.

Wheeler's Handbook of Medicine. By Wm. R. Jack, M.D. Fourth Edition. (Edinburgh: E. and S. Livingstone. 1912. Price 8s.)

This handbook is well known to all Conjoint students, and they have found it one of the most useful and handy volumes for revision purposes. The present edition, again edited by Dr. Jack, embodies many modifications necessitated by the recent advances which have been made in medicine. The combination of new matter with the thoroughness and excellence of the old framework makes *Wheeler* still one of the best books for the student who wishes somewhat more detail and greater fulness than the ordinary "cram" synopsis can give him. Undoubtedly one of the most difficult subjects for the average student is the diseases of the nervous system; while it is, of course, impossible for anyone to hope to master this subject without taking pains and without, as a preliminary, fully acquainting himself with the elementary anatomy and physiology of the regions studied, yet a good text-book may give considerable help even to the painstaking student, and conversely an ill-written manual, however full and complete, may render the subject so inchoate and obscure that no student can be expected to make headway. In *Wheeler* this subject of the diseases of the nervous system and the collateral one of the dystrophies are treated in a concise and perfectly clear manner. We may instance the paragraphs dealing with the cranial nerves, especially the third, fourth, and sixth nerves, the lesions of which always present great difficulties to the student. Equally good is the description of valvular diseases; it is preceded by an excellent summary on cardiac sounds. Here and there the descriptions are not up to the standard set in these two groups which we have alluded to. For instance, the pathology of the condition known as duodenal ulcer is very scantily treated; again, constipation is dismissed in little more than a page, while the treatment is compressed into a small paragraph, with the conclusion of which most examiners will probably join issue. In the appendicitis section it is stated that immediate operation is now the received practice; recent correspondence in a contemporary has shown that the truth of this desirable dogma is not so generally accepted as it should be. Possibly, in view of the conservatism of some examiners, the student may find it desirable to hedge by putting a conditional clause in his answer. We have, however, no doubt that this handy manual will still remain a great favourite for many years to come.

